# The Association between Gly460Trp-Polymorphism of Alpha-Adducin 1 Gene (*ADD1*) and Arterial Hypertension Development in Ukrainian Population

**DOI:** 10.1155/2021/5596974

**Published:** 2021-05-04

**Authors:** Svitlana Yermolenko, Yaroslav Chumachenko, Viktor Orlovskyi, Irina Moiseyenko, Oleksandr Orlovskyi

**Affiliations:** ^1^Department of Family Medicine, Medical Institute of the Sumy State University, Sumy 40035, Ukraine; ^2^Scientific Laboratory of Molecular Genetic Research, Medical Institute of the Sumy State University, Sumy 40035, Ukraine

## Abstract

Arterial hypertension (AH) belongs to the diseases with genetic predisposition that determines the necessity of research on the genetic component's influence on this disease development. It is suggested that one of the salt-sensitive arterial hypertension potential markers may be the alpha-adducin gene because its protein product is involved in the ion transport regulation in the renal epithelium. Thus, the aim of the study was to investigate the association between *ADD1* Gly460Trp-polymorphism and the AH development risk among patients with different risk factors in the Ukrainian population. The study included 232 Ukrainians: 120 patients with diagnosed arterial hypertension and 112 practically healthy individuals. Polymerase chain reaction-restriction fragment length polymorphism (PCR-RFLP) analysis was used for *ADD1* Gly460Trp-polymorphism genotyping. The *ADD1* Gly460Trp-polymorphic locus is an important predictor of arterial hypertension development in the Ukrainian population, but other nongenetic factors should be considered in further studies.

## 1. Introduction

Arterial hypertension (AH) is the leading preventable risk factor of cardiovascular diseases (CVDs). Its prevalence is about 30–45% of the total population and increases with age. High arterial blood pressure (BP) is known to provoke and accelerate the development of atherosclerosis, which significantly increases the incidence of coronary heart disease, stroke, heart, and kidney failure [1].

Arterial hypertension is a disease with a genetic predisposition, whose development depends on the complex interaction of environmental factors and genetic markers that influence the individual risk of disease occurrence, its course, and the complications formation. There are nonmodifiable (sex, age, and burdened heredity) and modifiable (overweight and obesity, smoking, alcohol consumption, high-salt intake, stress, etc.) AH risk factors [2].

Genetic factors play an important role in AH development. Therefore, there are a lot of studies that focused on the AH risk genes detection, whose expression products can directly or indirectly affect the AH occurrence and the clinical manifestations of pathogenetically related CVD [3–5]. There is a separate area of research devoted to the study of the gene single nucleotide polymorphisms (SNP) role, whose products are important in the AH pathogenesis [4, 6].

Present genetic epidemiological studies suggest that the adducin gene may be an important potential marker gene for AH, especially for salt-sensitive [7]. Today, there are 3 major loci in the adducin family genes that correlate with the AH development–Gly460Trp *ADD1*, C1797T *ADD2*, and A386G *ADD3* [8, 9].

A number of population studies revealed an association between the Gly460Trp T allele (*ADD1* gene) and AH development [10–18]; however, in others, this link is absent [19–22]. It should be noted that data on the association between *ADD1* Gly460Trp-polymorphism and the AH occurrence remain controversial and demonstrate the interpopulation differences in the association of gene polymorphic variants with the risk of AH development. Many studies have shown that differences in genotype distribution can significantly fluctuate in various ethnic groups [10–22]. Therefore, the association between these or other polymorphic markers and the AH development is contradictory in different populations, making research for each ethnic and population group significant [8, 22–24].

The purpose of this work is to investigate the association between Gly460Trp-polymorphism of the alpha-adducin 1 (*ADD1*) gene and the AH development risk among patients with different risk factors in the Ukrainian population.

## 2. Materials and Methods

### 2.1. Study Population

The present hospital-based case-control study enrolled 232 Ukrainians that had been under observation in Sumy Regional Hospital and 1th Sumy Clinical Hospital from 2018 to 2019. All participants were selected from the medical records and divided into case groups with diagnosed AH and a control group of relatively healthy controls without AH. The first group involved 120 patients (46 males and 74 females; mean age (±SD) 53.33 ± 14.55) with a verified diagnosis of AH II clinical stage. Verification of diagnosis and determination of AH stage and grade were estimated according to WHO criteria (1999) [25] and European Society of Cardiology (2007) [26]. The control group included 112 practically healthy normotensive individuals (73 males and 39 females; mean age 57.89 ± 10.05). Exclusion criteria from the study group were the symptomatic AH, endocrine obesity, pregnancy and lactation, acute infectious diseases, exacerbation of chronic infectious diseases, neoplasms, mental disorders and nervous system diseases, systemic connective tissue diseases, acute disorders of cerebral circulation (up to 3 months), and the patient's refusal to participate in the research.

At the next stage, patients with hypertension were divided into two subgroups—with a salt-sensitive and salt-resistant course of the disease (Table 1). Elijovich technique was used to determine the type of salt sensitivity. The essence of this method is to stick to a high-salt diet with a further restriction on salt intake. Sticking to a diet was evaluated by daily urinary sodium excretion during high-salt and low-salt diets using electrolyte analyzer “Ionometer3” (Fresenius Medical Care AG and Co. KGaA, Germany) for ion-selective urine potentiometry. There was a characteristic of dependence on the salt diet: patients were referred to salt-sensitive if after switching from high-salt to low-salt diets, systolic blood pressure (SBP) decreased by more than 10 mmHg; to salt-resistant if after changing diets, SBP did not decrease by more than 5 mmHg; and individuals with a paradoxical reaction to the change in salt load and a decrease in SBP during the switching to a low-salt diet [27].

Anthropometric data (age, sex, BMI, and smoking habit) was recorded at the time of selection to the study. BP was measured on three occasions, and the mean of the last two was used in the analysis. Fasting glucose and lipid profile parameters (total cholesterol, triglyceride, high, low, and very low-density lipoprotein cholesterol, and atherogenic index of plasma) were recorded at the time of diagnosis. Daily urinary sodium excretion was performed for patients with diagnosed AH to determine the type of salt sensitivity.

The study protocol was implemented in accordance with the Declaration of Helsinki and was approved by the Ethics Committee of the Medical Institute of Sumy State University. Patients were introduced to the study protocol and they gave a voluntary informed written consent.

### 2.2. Genotyping

*ADD1* Gly460Trp-polymorphism determination was performed using the polymerase chain reaction-restriction fragment length polymorphism analysis (PCR-RFLP). DNA was isolated from 50 *µ*L of whole venous blood according to the manufacturer's standard protocol using «NeoPrep^100^DNA_Blood» (Neogene). A reaction mixture for the amplification (total volume 25 *µ*L) was composed of 5 *µ*L FastDigest Green Buffer (10X) (Thermo Scientific™, USA), 0.5 *µ*L dNTP Mix (containing 10 mM of each deoxyribonucleotide) (Thermo Scientific™, USA), 0.75 U DreamTaq DNA Polymerase (5 U/*µ*L) (Thermo Scientific™, USA), 0.1 *μ*l of each primer, 2 *µ*L DNA, and deionized water to the total volume. PCR was carried out using the Thermocycler GeneAmp PCR system 2700 (Thermo Fisher Scientific, USA). The primer nucleotide structure and PCR stages are shown in Table 2.

The reaction mixture (total volume 2 *µ*L) for restriction included 0.8 *µ*L CutSmart Buffer (New England BioLabs), 0.2 *µ*L *Sau*96I (New England BioLabs), and deionized water to the total volume. Samples were incubated in a thermostat at 37°С for 20 h.

**Figure 1:**
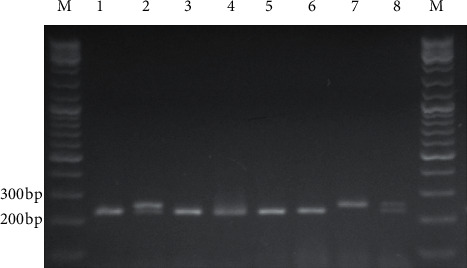
Results of the *ADD1* rs4961-polymorphism genotyping. M: molecular marker; bp: base pairs; 1, 3, 4, 5, 6: GG genotype; 2, 8: GT genotype; 7: ТТ genotype.

Horizontal electrophoresis (10 V/cm) in 2.5% agarose gel with the addition of a bromide ethidium solution (10 mg/ml) was used for genotyping. Discrimination of genotypes was carried out using a transilluminator (“Biocom”, Russia). The presence of the G allele at the 66124th position of the *ADD1* gene (NG_012037.1) led to the splitting of the amplicon (252 bp) by *Sau*96I-restrictase into two parts of 225 bp and 27 bp. The presence of T allele resulted in preventing the restriction, and the original fragment (252 bp) was preserved (Figure 1).

### 2.3. Statistical Analysis

The Statistical Package for Social Science software (SPSS, version 25.0, Chicago, IL, USA) was applied for statistical analysis of the obtained results. Continuous variables were presented as the mean ± SD, and categorical variables as absolute and percentage values. Chi-square (*χ*^2^) test was used for comparing the frequency of alleles, genotypes, and other categorical variables. A two-tailed Student's *t*-test was used to compare the mean values (with preliminary verification of the data distribution for normality according to the Shapiro-Wilk test). The correspondence of the allele frequency distribution to Hardy-Weinberg equilibrium was carried out using the Calculator of Hardy–Weinberg equilibrium (https://wpcalc.com/en/equilibrium-hardy-weinberg). The binary logistic regression was used in the framework of recessive, dominant, overdominant, and additive inheritance models to analyze the association between the *ADD1* rs4961-polymorphic variant and AH development. Adjustments for age, sex, body mass index (BMI), smoking habit, AH grade, and the AH presence in family history were applied to improve the reliability of the obtained results. «Sex» and «BMI» covariates were explored as effect modifiers by adding the «model of inheritance∗sex» and «model of inheritance∗BMI» independent variables to the logistic regression, respectively. All *P* values are two-tailed; a value of *P* < 0.05 was accepted as significant.

## 3. Results

Statistically significant differences were revealed between salt-sensitive and salt-resistant patients in sex (*P*=0.004) and BMI (*P* < 0.001). On the other hand, the comparison subgroups did not significantly differ in age, number of smokers, and laboratory test results (*P* > 0.05) (Table 1).

The frequency of *ADD1* Gly460Trp genotypes and alleles and correspondence of the distribution of the major and minor alleles to Hardy–Weinberg equilibrium are summarized in Table 3. Both the control and main groups did not significantly deviate from Hardy–Weinberg equilibrium expectations (*P*_HWE_ = 0.452 and *P*_HWE_ = 0.494, respectively).

The distribution of *ADD1* Gly460Trp genotypes and alleles analysis indicated that among 120 AH patients, 91 (75.8%) had the G allele in homozygosis, 3 (2.5 %) had the T allele in homozygosis, and 26 (21.7 %) were heterozygous. The frequency of AH patients' G allele was 0.87, and *T* was 0.13. And, 98 (87.5 %) had GG genotype, 13 (11.6 %) had GT genotype, and 1 (0.9 %) had TT genotype among practically healthy individuals. The *G* and *T* frequencies were 0.93 and 0.07, respectively. Chi-square (*χ*^2^) test did not reveal a distinction in the distribution of *ADD1* Gly460Trp genotypes and alleles in control and AH patients (*P*=0.07); instead, statistically significant differences in the allele frequency were established (*P*=0.033).

A binary logistic regression was used to estimate the AH occurrence risk depending on the *ADD1* Gly460Trp genotypes (Table 4). An AH development risk for minor allele carriers (GT + TT) is higher (OR_с_ = 2.231; 95 % СІ = 1.109–4.487) than that for a major allele in homozygosis (GG) (*Р*_с_ = 0.024). Also, GT genotype has higher AH development risk than GG genotype under overdominant and additive models of inheritance (OR_с_ = 2.106; 95 % СІ = 1.022–4.341; *Р*_с_ = 0.043 for overdominant; OR_с_ = 2.154; 95 % СІ = 1.044–4.444; *Р*_с_ = 0.038 for additive). After adjusting for the covariates (age, BMI, and smoking habit), a significant association remained under the dominant model (OR_а_ = 2.138; 95 % СІ = 1.014–4.509; *Р*_а_ = 0.046). However, after adjusting for the sex, the dominant model lost its statistical significance (*Р*_а_ = 0.261).

The next stage was to study the Gly460Trp-polymorphism genotypes distribution, considering some AH risk factors.

Statistically significant differences were found in the distribution of genotypes (*P*=0.002) and alleles (*P* < 0.001) among individuals of different sexes (Table 5). Thus, 48 (64.8%) subjects with GG genotype, 23 (31.1%) with GT genotype, and 3 (4.1%) with TT genotype were among females with AH; minor allele frequency was 0.19. Also, there were 43 (93.5 %) males with GG genotype and 3 (6.5 %) with GT genotype; minor allele frequency was 0.03 (there were no males with TT genotype).

AH patients were divided into groups with normal and elevated BMI to reveal significant differences in the genotype distribution in individuals with different BMIs (*P*=0.008) (Table 5). Thus, 40 (64.5 %) subjects with GG genotype, 19 (30.7 %) with GT genotype, and 3 (4.8 %) with TT genotype were among AH patients with BMI ≥ 25 kg/m^2^. AH patients with BMI ˂ 25 kg/m^2^ had the following distribution of GG, GT, and TT genotypes: 51 (87.9 %), 7 (12.1 %), and 0 (0 %), respectively. The minor allele frequency in the first group was 0.2 and 0.06 in the second group which had statistically significant differences in its distribution (*P*=0.001).

The distribution of G/G, G/T, and T/T genotypes in salt-sensitive patients significantly differed from salt-resistant patients (*P*=0.022) (Table 5). The genotype distribution was 41 (66.2 %), 18 (29 %), and 3 (4.8%) for salt-sensitive patients and 50 (86.2 %), 8 (13.8 %), and 0 (0 %) for salt-resistant patients, respectively. The minor allele frequency in the first group was 0.19 and 0.07 in the second group which had statistically significant differences in its distribution (*P*=0.005).

There were no statistically significant differences in *ADD1* Gly460Trp genotypes (*P*=0.069) and minor allele (*P*=0.424) distributions among subjects with smoking habits.

A binary logistic regression was used to estimate the AH occurrence risk among patients of different sexes depending on the *ADD1* Gly460Trp genotypes (Table 6). There was no statistically significant AH development risk in different genotypes under each inheritance model (*P*_c_ > 0.05). However, after adjusting for covariates (age, BMI, and smoking habit), a significant link was revealed under the dominant model in females (OR_а_ = 2.787; 95 % СІ = 1.022–7.6; *Р*_а_ = 0.045). Thus, sex can be defined as a risk modifier for AH development (*P*_a_^int^ = 0.049). It confirms the view that the AH development risk is higher in females with the T allele presence.

After regression analysis, the risk of AH development in patients with different *ADD1* Gly460Trp genotypes was determined depending on BMI (Table 7). A significant association between *ADD1* rs4961-polymorphic variant and AH was revealed for the patients with BMI ≥25 kg/m^2^ under all models of inheritance. The minor T allele (GT + TT) carriers were found to have a higher AH development risk than *G* allele (GG) carriers (*Р*_с_ = 0.001) under the dominant model (OR_с_ = 4.408; 95 % СІ = 1.859–10.454). Also, GT genotype has a significantly higher AH development risk than GG genotype under overdominant (OR_с_ = 4.07; 95 % СІ = 1.649–10.046; *Р*_с_ = 0.002) and additive (OR_с_ = 4.312; 95 % СІ = 1.74–10.689; *Р*_с_ = 0.002) models. After adjusting for covariates (age and smoking habit), a significant association remained under each model of inheritance (OR_а_ = 3.527; 95 % СІ = 1.442–8.625; *Р*_а_ = 0.006 for the dominant model; OR_а_ = 3.418; 95 % СІ = 1.342–8.704; *Р*_а_ = 0.001 for the overdominant model; OR_а_ = 3.582; 95 % СІ = 1.402–9.154; *Р*_а_ = 0.008 for additive model). It should be noted that BMI is a risk modifier for AH development both before (*P*_c_^int^ = 0.017; *P*_c_^int^ = 0.025; *P*_c_^int^ = 0.021, respectively) and after adjustment for covariates (*P*_a_^int^ = 0.027; *P*_a_^int^ = 0.032; *P*_a_^int^ = 0.028, respectively). However, there was no significant association after adjusting for sex under all models of inheritance (*Р*_а_ > 0.05 та *P*_a_^int^ > 0.05).

The assessment of salt-sensitive AH development risk depending on the *ADD1* Gly460Trp genotype is shown in Table 8. The minor T allele (GT + TT) carriers were found to have a higher salt-sensitive AH development risk than G allele (GG) carriers (*Р*_с_ = 0.013) under the dominant model of inheritance (OR_с_ = 3.201; 95 % СІ = 1.285–7.977). Also, GT genotype has a significantly higher salt-sensitivity development risk than GG genotype under overdominant (OR_с_ = 2.557; 95 % СІ = 1.013–6.455; *Р*_с_ = 0.047) and additive (OR_с_ = 2.774; 95 % СІ = 1.083–6.952; *Р*_с_ = 0.033) models. After adjusting for covariates (age, smoking habit, AH grade, and AH presence in the family history), a significant association remained under dominant (OR_а_ = 3.141; 95 % СІ = 1.221–8.08; *Р*_а_ = 0.018) and additive (OR_а_ = 2.756; 95 % СІ = 1.056–7.196; *Р*_а_ = 0.038) models. After adjusting for sex and BMI, a significant association was lost under all models of inheritance (*Р*_а_ > 0.05).

The next analysis stage was to study the distribution of genotypes and alleles in AH patients of different sexes and salt-sensitivity development risk assessment. The distribution of *ADD1* Gly460Trp-polymorphic variants is presented in Table 9. The GG, GT, and TT genotype distribution in the salt-sensitive female group was 25 (54.4 %), 18 (39.1 %), and 3 (6.5 %), while that in the salt-resistant female group was 23 (82.1 %), 5 (17.9 %), and 0 (0 %), respectively. There were statistically significant differences in both genotype (*P*=0.04) and allele (*P*=0.011) distributions in these comparison groups. In males, significant differences in genotype and allele distributions were not revealed (*P* > 0.05).

Binary logistic regression analysis showed that the presence of minor T allele (GT- and TT genotype) has a higher risk of salt-sensitivity development in females than GG genotype (OR_с_ = 3.864; 95 % СІ = 1.251–11.935; *Р*_с_ = 0.019). Also, the GT genotype has a significantly higher salt-sensitivity development risk than GG genotype under additive model (OR_с_ = 3.312; 95 % СІ = 1.058–10.369; *Р*_с_ = 0.04). After adjusting for covariates (age, smoking habit, AH grade, and AH presence in the family history), a statistically significant link remained under dominant (OR_а_ = 5.213; 95 % СІ = 1.36–19.99; *Р*_а_ = 0.016) and additive (OR_а_ = 4.445; 95 % СІ = 1.151–17.165; *Р*_а_ = 0.03) models of inheritance (Table 10). However, there was no significant association after adjusting for BMI under all models of inheritance (*Р*_а_ > 0.05).

## 4. Discussion

Adducin is a heterodimeric protein of the cell cytoskeleton that consists of 3 subunits (*α*, *ß*, *γ*). Alpha- and gamma-adducins are presented in almost all tissues, whereas beta-adducin is highly expressed in brain tissues and located in the erythrocyte membrane as a cytoplasmic protein. Adducin has various important types in physiological processes. Alpha-adducin is a major membrane ion transport regulator. It promotes spectrin to actin attachment, is able to bind to calmodulin, a substrate for protein kinases C and A, and regulates the Na^+^-K^+^-ATPase activity (transports sodium and potassium ions through the renal epithelium membrane). The structural feature of alpha- and beta-adducins is the presence of protease-resistant N-terminal region and protease-sensitive hydrophilic C-terminal region in their molecules [15, 28].

The various protein subunits are encoded by different genes (*ADD1*, *ADD2*, and *ADD3*) on three different chromosomes. Alternative splicing determines the existence of different adducin isoforms; however, not all variants have been fully described for today. The adducin *α*-subunit gene (*ADD1*) (NCBI) is located at the short arm of the 4th chromosome in the 16.3 segment. It consists of 16 exons (from 34 to 1892 bp) [29, 30].

Gly460Trp-polymorphism (rs4961) is the most studied and functionally important *ADD1*gene polymorphic site. The essence of rs4961-polymorphism is the replacement of guanine by thymine at the 1378th position in ADD1 mRNA (NM_001119.4). In turn, it leads to the replacement of glycine by tryptophan in the 460th position of the polypeptide chain [24].

Cusi et al. for the first time established the association between *ADD1* Gly460Trp-polymorphism and AH development for the French and Italians [10]. In 1998, consistent data were obtained by S. Tamaki et al. for the Japanese. Later, Nakamura et al. conducted a large-scale study involving an abundant amount of patients to study 4 genetic AH predictors. The authors concluded that *ADD1* Gly460Trp-polymorphism is an independent genetic marker for AH occurrence in the Japanese population [12]. An association between T allele and AH development was revealed among Russians [15], Chinese [14], Japanese [11], inhabitants of Madeira [18], Tunisia [16], North Africa [13], and Asian population [17]. The results we have obtained also indicate an association between *ADD1* Gly460Trp-polymorphism and AH occurrence in Ukrainians and demonstrate the higher T allele frequency in patients with salt-sensitive AH. However, it should be noted that after adjusting for covariates, a statistically significant link remained only in the female subgroup. The minor T allele carriers were revealed to have a higher AH development risk.

Howsoever, the association between *ADD1* Gly460Trp-polymorphism and AH occurrence was not found in patients with essential AH, namely, the inhabitants of India [22], Korea [21], Japan [19], and America [20].

Investigating the possible *ADD1* role as an AH genetic marker has practical importance. Cusi et al. [10] in their first works have already shown a higher T allele frequency in patients with salt-sensitive AH. They also described the association between this polymorphism and high blood pressure and its decrease after salt intake reducing. In 2002, Psaty et al. [31] and, later, Citterio et al. [32] described that T allele patients (GT and ТТ genotypes) differ in a significant blood pressure decrease after hydrochlorothiazide diuretics application. We have obtained comparable results regarding the protective minor T allele value in the salt-resistant AH development. However, it should be noted that sex and BMI are also important factors that determine the sensitivity to salt intake reducing in patients with high blood pressure.

The adducin gene regulates blood pressure, mainly by affecting the Na^+^-K^+^-ATPase activity and altering the sodium reabsorption by the renal tubules [30]. *In vivo* adducin is a substrate for PKC, PKA, and Rho-associated kinase [28] and is involved in cell signal transduction. Besides, adducin interacts with other membrane skeleton components and membrane proteins to influence ion transport, particularly, Na^+^ transport (Na^+^ channels, Na^+^-H^+^ exchange, Na^+^-Li^+^ countertransport, and Na^+^-K^+^-Cl^−^ cotransport) [33, 34]. *α*- or *β*-adducin point mutations can lead to hypertension, as the phosphorylation pattern changes from tyrosine kinase to the PKA site. The mutated *α*-adducin variants have been shown to interact with the Src-SH2 domain, increasing Src activity and phosphorylation and activity of Src-dependent Na^+^-K^+^- ATPase [35].

Several studies have approved the blood pressure normalization in hypertensive rats under the rostafuroxin influence (antihypertensive drug) that suppressed the *α*-adducin and Src-SH2 domain interaction and disrupted the Src activation and Na^+^-K^+^-ATPase phosphorylation [36]. The association between *ADD1* Gly460Trp-polymorphism and the response to rostafuroxin treatment has been shown in clinical studies of the Chiara Lanzani group [37].

There is a high scientific community interest in the *ADD1* participation in AH occurrence. Several meta-analyses have been recently performed to study the association between hypertension risk and genetic polymorphisms. In 2010, Liu et al. [8] and Ramu et al. [22] reported on the lack of association between the *α*-adducin Gly460Trp-polymorphism and AH. At the same time, Liao et al. [23] and Jin et al. [24] support the hypothesis that T allele carriers have a higher AH developing risk in Asian populations. The results' ambiguity can be explained by several reasons. First is the AH heterogeneity and its ethnic characteristics. A large number of the studies demonstrated a link between Gly460Trp-polymorphic variants and AH for the Asian population, while data for other populations differ. Secondly, hypertension is a multifactorial disease, and most studies do not consider environmental factors (geographical location, climate, diet, and lifestyle) that are also associated with hypertension risk. In addition, one of the potential limitations may be small sample sizes and the inability to include all current research into meta-analyses.

On the one hand, *ADD1* is an important AH gene candidate, and on the other hand, the obtained data are controversial. Our results show that gender is an important modifier of the rs4961 *ADD1* genotype effect on AH development. A previous study established the insignificant sex by genotype interaction for rs4961 in an investigated white population. However, researchers have found that Gly460Trp polymorphism in interaction with C1797T polymorphism of *ADD2* increases the risk of hypertension in all women (adjusted relative risk, RR = 2.35, *P*=0.01) and postmenopausal subjects (RR = 2.92, *P*=0.03), but not in men. Moreover, women with T alleles in both rs4961 and C1797 loci had a significantly higher plasma renin activity relative to CC carriers, whereas the opposite effect was observed in men. Thus, the authors hypothesized that the sexual differences in rs4961 influence on AH development may be associated with the renin-angiotensin-aldosterone system [38].

Another potential mechanism to explain such observations is the existence of gender dimorphism in the CpG methylation pattern distribution along the *ADD1* promoter region. It was found for the Chinese population that *ADD1* CpG methylation level was associated with essential hypertension (EH) with a significantly higher methylation degree in females than in males. Further analysis showed that a lower methylation level of CpG1 dinucleotide was linked to increased EH risk among females, but not males. In contrast, the lower degree of CpG2-5 methylation was associated with higher EH risk exclusively among males [39]. The comparable results were received in recent research for Egyptians. The authors hypothesized that a lower level of *ADD1* CpG methylation causes a higher expression of *α*-adducin and Na^+^-K^+^-ATPase which leads to more intense Na^+^ reabsorption and EH development, consequently [40]. Thus, further studies are needed to confirm these hypotheses.

It is worth noting that the study has some limitations. The comparison groups should be expanded to confirm the results. It should be noted that the adjustment for only three variables (age, BMI, and smoking) was used in regression analysis. Moreover, along with the Gly460Trp-polymorphic locus influence on the AH development, the importance of other, nongenetic, factors (sex, BMI, etc.) has been established. It is further important to determine the *ADD1* gene expression level, depending on its polymorphic variant, which is necessary for the Gly460Trp-polymorphism functional value establishing. Due to the existence of gender dimorphism, it will be necessary to estimate the interaction effect of rs4961 and C1797 SNP on AH development as well as investigate the methylation level of CpG islands in the *ADD1* promoter region.

## 5. Conclusions

Thus, *ADD1* Gly460Trp-polymorphism was found to increase the AH risk and the salt-sensitive AH development among the Ukrainian population. Further studies are necessary to confirm the obtained results and to establish the other nongenetic factors' role in the AH development, depending on the Gly460Trp-polymorphism genotype.

## Figures and Tables

**Table 1:** Clinical characteristics of the patients with various AH forms.

Parameter	SS	SR	*P*
	(*n* = 62)	(*n* = 58)	
Age, years	51.44 ± 14.91	55.36 ± 13.99	0.14
Sex, female/male	16/46	30/28	0.004
BMI, kg/m^2^	29.72 ± 4.72	25.17 ± 3.33	˂0.001
Smokers, *n* (%)	10 (16.1)	11 (19)	0.683
Sodium, urine (І phase)	20.11 ± 4.85	19.78 ± 4.54	0.696
Sodium, urine (ІІ phase)	203.1 ± 12.17	205.0 ± 11.88	0.388
Glucose, mmol/L	4.55 ± 0.63	4.57 ± 0.67	0.86
Total cholesterol, mmol/L	4.48 ± 1.25	4.36 ± 1.06	0.583
Triglyceride, mmol/L	1.54 ± 0.92	1.38 ± 0.79	0.3
HDL cholesterol, mmol/L	1.08 ± 0.17	1.07 ± 0.13	0.689
LDL cholesterol, mmol/L	2.7 ± 1.04	2.67 ± 0.96	0.861
VLDL cholesterol, mmol/L	0.65 ± 0.31	0.63 ± 0.35	0.859
AIP	3.33 ± 1.6	3.18 ± 1.33	0.583

*n*: number of cases; AH: arterial hypertension; SS: salt-sensitive; SR: salt-resistant; BMI: body mass index; HDL: high-density lipoprotein; LDL: low-density lipoprotein; VLDL: very low-density lipoprotein; AIP: atherogenic index of plasma.

**Table 2:** Primer nucleotide sequence and PCR conditions.

Primer nucleotide sequence	PCR conditions (*n* = 40 cycles)			PCR amplicon size	Restriction fragments
	D	H	E		
Fwd: 5`GACCCTAGGGCTACAGAACTG3`	94°C (30 s)	63°C (30 s)	72°C (30 s)	252	GG 225; 27
					GT 252; 225; 27
Rev: 5`TCGACTTGGGACTGCTTCCATTCGGCC3`					
					TT 252

D: denaturation; H: hybridization; E: elongation; Fwd: forward primer; Rev: reverse primer.

**Table 3:** Distribution of the *ADD1* rs4961-polymorphism alleles and genotypes in comparison groups.

	AH		Control	
	(*n* = 120)		(*n* = 112)	
	n	%	n	%
	Genotypes			
GG	91	75.8	98	87.5
GT	26	21.7	13	11.6
TT	3	2.5	1	0.9
P	0.07			

	Alleles			
G	224	87.5	209	93.3
T	32	12.5	15	6.7
P	0.033			
*P*_HWE_	0.494		0.452	

AH: arterial hypertension; *n*: number of cases; *P*_*HWE*_: *P* value of Hardy–Weinberg equilibrium.

**Table 4:** Analysis of the *ADD1* rs4961-polymorphism association with arterial hypertension occurrence.

Model of inheritance	*P*_*c*_	OR_c_ (95% CI)	*P*_*a*_^*1*^	OR_a_ (95% CI)
Dominant	0.024	2.231 (1.109–4.487)	0.046 (0.261)	2.138 (1.014–4.509)
Recessive	0.368	2.846 (0.292–27.711)	0.475 (0.541)	2.32 (0.23–23.413)
Overdominant	0.043	2.106 (1.022–4.341)	0.072 (0.352)	2.023 (0.939–4.359)
Additive^а^	0.038	2.154 (1.044–4.444)	0.061 (0.299)	2.087 (0.966–4.51)
	0.314	3.231 (0.33–31.621)	0.349 (0.441)	3.034 (0.297–30.993)

*P*_*c*_: crude *P* value; OR_c_: crude odds ratio; *P*_*a*_: *P* value adjusted for age, BMI, and smoking habit; OR_a_: odds ratio adjusted for age, BMI, and smoking habit; 95% CI: 95% confidence interval. a: upper row: comparison between GT and TT genotypes; lower row: comparison between TT and GG genotypes. 1: *P* value adjusted for sex is shown in parentheses.

**Table 5:** The *ADD1* rs4961-polymorphism allelic variants frequency in subjects with different AH risk factors.

Risk factors	Genotypes				
	G/G, *n* (%)	G/T, *n* (%)	T/T, *n* (%)	G, *n* (%)	T, *n* (%)
Males	43 (93.5)	3 (6.5)	0 (0)	89 (96.7)	3 (3.3)
Females	48 (64.8)	23 (31.1)	3 (4.1)	119 (80.4)	29 (19.6)
	Р = 0.002			Р < 0.001	

BMI < 25	51 (87.9)	7 (12.1)	0 (0)	109 (94)	7 (6)
BMI ≥ 25	40 (64.5)	19 (30.7)	3 (4.8)	99 (79.8)	25(20.2)
	Р = 0.008			Р = 0.001	

Nonsmoker	74 (74.8)	22 (22.2)	3 (3)	170 (85.9)	28 (14.1)
Smoker	17 (81)	4 (19)	0 (0)	38 (90.5)	4 (9.5)
	Р = 0.669			Р = 0.424	

Salt-resistant	50 (86.2)	8 (13.8)	0 (0)	108 (93.1)	8 (6.9)
Salt-sensitive	41 (66.2)	18 (29)	3 (4.8)	100 (80.6)	24 (19.4)
	Р = 0.022			Р = 0.005	

*n*: number of cases; BMI: body mass index.

**Table 6:** Association analysis between *ADD1* rs4961-polymorphism and arterial hypertension development among individuals of different sexes.

Model of inheritance^1^	*P*_*c*_	OR_c_ (95% CI)	*P*_c_^int^	*P*_*a*_	OR_a_ (95% CI)	*P*_a_^int^
Dominant	0.559	0.658 (0.161–2.684)	0.125	0.295	0.446 (0.098–2.023)	0.049
	0.061	2.476 (0.961–6.383)		0.045	2.787 (1.022–7.6)	

Overdominant	0.734	0.779 (0.185–3.281)	0.269	0.402	0.513 (0.108–2.437)	0.116
	0.137	2.062 (0.794–5.355)		0.105	2.299 (0.839–6.3)	

Additive^2^	0.718	0.767 (0.182–3.233)	0.234	0.388	0.504 (0.106–2.39)	0.095
	0.108	2.19 (0.841–5.704)		0.079	2.483 (0.902–6.839)	

*P*_*c*_: crude *P* value; OR_c_: crude odds ratio; P_c_^int^: crude *P* value interaction terms; *P*_*a*_: *P* value adjusted for age, BMI, and smoking habit; OR_a_odds ratio adjusted for age, BMI, and smoking habit; P_a_^int^: *P* value interaction terms adjusted for covariates; 95% CI: 95% confidence interval. 1: upper row: results for males; lower row: results for females. 2: upper row: comparison between GT and TT genotypes; lower row: comparison between TT and GG genotypes.

**Table 7:** Analysis of the association between *ADD1* rs4961-polymorphism and arterial hypertension occurrence according to BMI.

Model of inheritance^1^	*P*_*c*_	OR_c_ (95% CI)	*P*_c_^int^	*P*_*a*_^*3*^	OR_a_ (95% CI)	*P*_a_^int3^
Dominant	0.556	0.682 (0.191–2.433)	0.017	0.407 (0.39)	0.564 (0.145–2.186)	0.027 (0.077)
	0.001	4.408 (1.859–10.454)		0.006 (0.063)	3.527 (1.442–8.625)	

Overdominant	0.556	0.682 (0.191–2.433)	0.025	0.405 (0.387)	0.561 (0.144–2.185)	0.032 (0.092)
	0.002	4.07 (1.649–10.046)		0.001 (0.094)	3.418 (1.342–8.704)	

Additive^2^	0.556	0.682 (0.191–2.433)	0.021	0.407 (0.392)	0.564 (0.145–2.187)	0.028 (0.079)
	0.002	4.312 (1.74–10.689)		0.008 (0.072)	3.582 (1.402–9.154)	

*P*_*c*_: crude *P* value; OR_c_ crude odds ratio; *P*_c_^int^crude *P* value interaction terms; *P*_*a*_*P* value adjusted for age and smoking habit; OR_a_odds ratio adjusted for age and smoking habit; P_a_^int^*P* value interaction terms adjusted for covariates; 95% CI 95% confidence interval. 1upper rowresults for BMI < 25 kg/m^2^; lower rowfor BMI ≥25 kg/m^2^. 2: upper row: comparison between GT and TT genotypes; lower row: comparison between TT and GG genotypes. 3*P* value adjusted for sex is shown in parentheses.

**Table 8:** Association analysis between *ADD1* rs4961-polymorphism and salt-sensitivity occurrence.

Model of inheritance	*P*_*c*_	OR_c_ (95% CI)	*P*_*a*_^*2*^	OR_a_ (95% CI)
Dominant	0.013	3.201 (1.285–7.977)	0.018 (0.443)	3.141 (1.221–8.08)
Overdominant	0.047	2.557 (1.013–6.455)	0.054 (0.574)	2.562 (0.983–6.675)
Additive^1^	0.033	2.774 (1.083–6.952)	0.038 (0.509)	2.756 (1.056–7.196)

*P*_*c*_crude *P* value; OR_c_crude odds ratio; *P*_*a*_*P* value adjusted for age, smoking habit, AH presence in family history, and AH grade; OR_a_ odds ratio adjusted for age, smoking habit, AH presence in family history, and AH grade; 95% CI95% confidence interval. 1upper row: comparison between GT and TT genotypes; lower row: comparison between TT and GG genotypes. 2: *P* value adjusted for sex and BMI is shown in parentheses.

**Table 9:** Distribution of the *ADD1* rs4961-polymorphism alleles and genotypes among males and females.

	SS		SR	
	n	%	n	%
	Males (*n* = 46)			
	Genotypes			
GG	16	100	27	90
GT	0	0	3	10
TT	0	0	0	0
P	0.191			
	Alleles			
G	32	100	57	95
T	0	0	3	5
P	0.198			

	Females (*n* = 74)			
	Genotypes			
GG	25	54.4	23	82.1
GT	18	39.1	5	17.9
ТТ	3	6.5	0	0
Р	0.04			
	Alleles			
G	68	73.9	51	91.1
T	24	26.1	5	8.9
P	0.011			

*n*: number of cases; SS: salt-sensitive; SR: salt-resistant.

**Table 10:** Association analysis between *ADD1* rs4961-polymorphism and salt-sensitivity occurrence among females.

Model of inheritance	*Р*_*с*_	OR_c_ (95% CI)	*P*_*a*_^*2*^	OR_a_ (95% CI)
Dominant	0.019	3.864 (1.251–11.935)	0.016 (0.258)	5.213 (1.36–19.99)
Overdominant	0.061	2.957 (0.951–9.191)	0.053 (0.377)	3.714 (0.983–14.03)
Additive^1^	0.04	3.312 (1.058–10.369)	0.03 (0.3)	4.445 (1.151–17.165)

*P*_*c*_crude *P* value; OR_c_crude odds ratio; *P*_*a*_*P* value adjusted for age, smoking habit, AH presence in family history, and AH grade; OR_a_odds ratio adjusted for age, smoking habit, AH presence in family history, and AH grade; 95% CI95% confidence interval. 1upper row: comparison between GT and TT genotypes; lower row: comparison between TT and GG genotypes. 2: *P* value adjusted for BMI is shown in parentheses.

## Data Availability

The data used to support the findings of this study are available from the corresponding author upon request.
